# Disordered Eating Behaviors and Psychological Distress in Patients with Type 2 Diabetes Mellitus: A Cross-Sectional Study from Tunisia

**DOI:** 10.3390/jcm15208115

**Published:** 2026-10-19

**Authors:** Rym Ben Othman, Syrine Trabelsi, Ismail Dergaa, Halil İbrahim Ceylan, Deniz Öztürk, Nicola Luigi Bragazzi, Yasmine Jallouli, Valentina Stefanica, Henda Jamoussi

**Affiliations:** 1 Research Unit on Obesity UR18ES01, Faculty of Medicine, University Tunis El Manar, 15 Rue Jebel Lakhdar, Beb Saadoun, Tunis 1007, Tunisia; benothmanr@gmail.com (R.B.O.); hendajamoussi@gmail.com (H.J.); 2 Faculty of Medicine, University of Tunis El Manar, Tunis, 11 Rue Jebel Lakhdar, Beb Saadoun, Tunis 1007, Tunisia; 3 National Institute of Nutrition and Food Technology of Tunis, 11 Jbal Lakhdar Street, Tunis 1007, Tunisia; syrinetr0@gmail.com (S.T.); follajallouli@gmail.com (Y.J.); 4 Higher Institute of Sport and Physical Education of Ksar Said, University of Manouba, Manouba 2010, Tunisia; phd.dergaa@gmail.com; 5 Physical Activity Research Unit, Sport and Health (UR18JS01), National Observatory of Sports, Tunis 1003, Tunisia; 6 Physical Education and Sports Teaching Department, Faculty of Sports Sciences, Atatürk University, Erzurum 25240, Türkiye; 7 Health Services Vocational School, Atatürk University, Erzurum 25240, Türkiye; d.ozturk@atauni.edu.tr; 8 Laboratory for Industrial and Applied Mathematics (LIAM), Department of Mathematics and Statistics, York University, Toronto, ON M3J 1P3, Canada; 9 Department of Physical Education and Sport, Faculty of Sciences, Physical Education and Informatics, National University of Science and Technology Politehnica Bucharest, Pitești University Center, 060042 Pitești, Romania; valentina.stefanica@upb.ro

**Keywords:** disordered eating behaviors, type 2 diabetes mellitus, anxiety, depression, psychological factors, treatment adherence, Tunisia

## Abstract

**Background**: Disordered eating behaviors (DEBs) and psychological distress may complicate type 2 diabetes mellitus (T2DM) care. **Objective**: To estimate the frequency of DEB screening positivity and examine associated characteristics in a Tunisian T2DM clinic sample. **Methods**: This cross-sectional study included 400 adults assessed between January and April 2023 using the Sick, Control, One stone, Fat, Food (SCOFF) questionnaire; Bulimic Investigatory Test Edinburgh (BITE); four-item Perceived Stress Scale (PSS-4); and Hospital Anxiety and Depression Scale (HADS). Exploratory analysis estimated crude prevalence ratios (PRs). Adjusted odds ratios (aORs) came from a logistic model including age, sex, body mass index (BMI), diabetes duration, and PSS-4. Separate secondary models replaced PSS-4 with the Hospital Anxiety and Depression Scale anxiety subscale (HADS-A) or depression subscale (HADS-D). **Results**: DEB positivity was 26.8% (107/400; 95% confidence interval [CI], 22.6–31.3%). Crude DEB prevalence was higher in women than men (30.4% versus 19.2%; PR, 1.58; 95% CI, 1.06–2.35), in participants with HADS-A ≥11 versus <11 (PR, 2.28; 95% CI, 1.64–3.17), and with HADS-D ≥11 versus <11 (PR, 1.49; 95% CI, 1.08–2.05). In the primary adjusted model, DEB positivity was associated with younger age (aOR per year, 0.952; 95% CI, 0.922–0.982), higher BMI (aOR per kg/m^2^, 1.083; 95% CI, 1.042–1.126), and higher PSS-4 (aOR per point, 1.133; 95% CI, 1.067–1.203). HADS scores were positively associated in separate adjusted models. Glycated hemoglobin (HbA1c) was high in both groups (10.36% versus 9.91%; difference, 0.45 percentage points; 95% CI, −0.01 to 0.92). **Conclusions**: Approximately one-quarter of this clinic sample had a positive DEB screen. Associations with psychological symptoms support attention to eating concerns and emotional well-being in diabetes care. However, cross-sectional data limit causal interpretation, and future longitudinal studies are warranted.

## 1. Introduction

Type 2 diabetes mellitus (T2DM) is a major public health concern worldwide, including in North Africa [1,2]. The 2021 International Diabetes Federation Atlas estimated that 537 million adults aged 20–79 years were living with diabetes and projected 783 million by 2045 [3]. A Tunisian national survey analysis described an increase in diabetes prevalence among adults aged 35–70 years from 13.1% in 1996 to 20.5% in 2016 [4]. A separate analysis of the 2016 Tunisian Health Examination Survey estimated an HbA1c-based prevalence of 18.2% [5]. Nutrition transition and socioeconomic differences in obesity provide relevant context for diabetes care in Tunisia [6,7,8]. Daily treatment, dietary decisions, and self-management also carry psychological demands [9].

Disordered eating behaviors (DEBs) encompass problematic eating attitudes and practices, including loss of control, binge eating, restrictive patterns, and compensatory behaviors. They may occur with or without a diagnosable eating disorder [10,11,12,13]. Diabetes management can interact with these behaviors, but relationships differ between diabetes types and across populations. Reviews focused on T2DM have identified binge eating as an important concern while noting heterogeneous prevalence estimates and assessment methods [14]. Evidence from insulin-treated populations also supports assessing diabetes-specific behaviors such as medication manipulation [15]. Associations with glycemic control are not uniform: some studies have found poorer control with disturbed eating, whereas others have not [16,17,18].

Much of the diabetes and eating-disorder literature concerns type 1 diabetes, particularly among girls and young women [19,20,21]. Findings from these populations cannot be assumed to apply to older adults with T2DM. Psychological models of eating disorders emphasize dietary restraint, weight and shape concerns, and emotional difficulties [22,23]. Weight stigma is another relevant consideration [24,25]. These frameworks can inform research questions, but their applicability to a Tunisian T2DM clinic population requires empirical evaluation.

The nutrition transition has changed food availability and dietary practices internationally and in the Eastern Mediterranean region [26,27,28]. Tunisian research has documented dietary and metabolic differences among people living with obesity, as well as substantial socioeconomic and sex differences in obesity [6,7,29]. Studies of female university students in Arab countries have examined media exposure, eating attitudes, and body image [30,31].

Diabetes distress, depression, and general perceived stress are related but distinct constructs [32]. Tunisian studies have documented diabetes distress and depressive symptoms among adults with T2DM [33,34]. Whether eating-related concerns accompany these psychological symptoms is clinically relevant, particularly where access to specialist mental health care may be limited. The present study aimed to estimate the frequency of a positive DEB screen in a Tunisian T2DM clinic sample, describe associated sociodemographic and metabolic characteristics, and examine associations with perceived stress, anxiety symptoms, and depressive symptoms.

## 2. Materials and Methods

### 2.1. Ethical Approval

This study received approval from the Ethics Committee of the National Institute of Nutrition of Tunis on 26 May 2022 (Approval reference: 10/2022). The research was conducted in accordance with the ethical principles outlined in the Declaration of Helsinki for medical research involving human subjects. All participants provided written informed consent after receiving detailed explanations about the study objectives, protocol, and their right to withdraw from the study at any time without consequences for their clinical care.

### 2.2. Sample Size Calculation

Sample size planning used the single-proportion formula:

$$n=Z^{2}p\frac{1-p}{d^{2}}$$

With a two-sided 95% confidence level (Z = 1.96), an anticipated proportion of 0.20, and an absolute precision of 0.04, the calculation yields 384.16, rounded up to 385 participants. The anticipated proportion is consistent with the 20% prevalence of eating disorders reported in a clinical T2DM sample by Papelbaum et al. [35], although clinical diagnoses and screening positivity are different outcomes.

### 2.3. Population

This cross-sectional study was conducted at the National Institute of Nutrition of Tunis, Tunis, Tunisia, between January and April 2023. The study protocol specified adults aged at least 40 years with confirmed T2DM according to American Diabetes Association criteria and diabetes duration exceeding one year. Exclusion criteria were schizophrenia, bipolar disorder, or intellectual disability affecting questionnaire completion; alcohol use disorders; psychotropic medication or corticosteroid use within the preceding two months; and conditions or physiological states that could affect appetite or eating behavior, including uncontrolled thyroid dysfunction, Cushing’s disease, acromegaly, adrenal insufficiency, type 1 or secondary diabetes, gestational diabetes, chronic kidney disease stages 4–5, active tuberculosis, human immunodeficiency virus infection, pregnancy, the first six postpartum months, and lactation. Of 541 individuals approached, 400 consented and met the reported eligibility criteria. Participants were recruited consecutively during outpatient follow-up visits and received no financial compensation.

### 2.4. Study Assessments and Outcome Definition

Each participant underwent a single assessment by a physician experienced in administering eating-disorder screening instruments. Sociodemographic information, medical history, lifestyle factors, and diabetes-related information were collected through a clinical interview and medical-record review. Anthropometric and fasting biochemical measurements were obtained, and participants completed Arabic-language SCOFF, BITE, PSS-4, and HADS questionnaires in a private clinical setting. DEB screening definition was SCOFF ≥ 2 and/or BITE symptom score ≥ 20.

### 2.5. Measurement Instruments

#### 2.5.1. Sick/Control/One Stone/Fat/Food (SCOFF) Questionnaire

SCOFF is a five-item screening questionnaire addressing vomiting, loss of control, weight loss, body image, and preoccupation with food [36]. A score of at least two positive responses warrants further assessment; however, it is not a diagnosis. An Arabic validation study in 123 women attending primary care in Beirut reported sensitivity of 80.0% and specificity of 72.7% at this threshold [37]. A broader systematic review reported pooled adult sensitivity of 84% and specificity of 80% [38].

#### 2.5.2. Bulimic Investigatory Test Edinburgh (BITE)

BITE is a 33-item self-report instrument developed to assess bulimic symptoms and their severity [39]. A symptom score of at least 20 indicates substantial bulimic symptomatology and a need for clinical assessment; it does not, on its own, establish either bulimia nervosa or binge eating disorder (BED). An Arabic validation study conducted in Tunisia was published in 2008 and included students and clinical eating-disorder groups [40].

#### 2.5.3. Perceived Stress Scale 4 (PSS-4)

The four-item Perceived Stress Scale (PSS-4) measures perceived unpredictability, lack of control, and stress over the previous month [41]. Responses are scored from 0 to 4, with positively worded items reverse-scored, yielding a total from 0 to 16. Higher totals indicate greater perceived stress; the scale has no diagnostic cutoff. Psychometric performance varies by setting. In a community sample of older adults, Ezzati et al. found satisfactory internal consistency for longer PSS versions [42].

#### 2.5.4. Hospital Anxiety and Depression Scale

HADS comprises seven anxiety items (HADS-A) and seven depression items (HADS-D), each scored from 0 to 3, giving subscale totals of 0–21 [43]. Arabic-language validation evidence is available [44], while the broader literature indicates that accuracy depends on the population and threshold [45]. Scores of 0–7, 8–10, and at least 11 conventionally indicate lower, borderline, and elevated symptom levels, respectively. They do not confirm psychiatric diagnoses.

#### 2.5.5. Anthropometric Measurements

Weight and height were measured using standardized techniques as described in the National Health and Nutrition Examination Survey (NHANES) anthropometry procedures manual. Weight was measured to the nearest 0.1 kg using a calibrated digital scale, with participants wearing light indoor clothing without shoes. Height was measured to the nearest 0.1 cm using a wall-mounted stadiometer, with participants standing barefoot in the Frankfurt plane position. Body mass index (BMI) was calculated using the formula: BMI = weight (kg)/[height (m)]^2^. Participants were classified according to World Health Organization (WHO) BMI categories: underweight (BMI < 18.5 kg/m^2^), normal weight (BMI 18.5 to 24.9 kg/m^2^), overweight (BMI 25.0 to 29.9 kg/m^2^), and obese (BMI ≥ 30.0 kg/m^2^).

#### 2.5.6. Biochemical Analyses

According to the study protocol, fasting blood was collected after at least eight hours, plasma glucose was measured by the hexokinase method, and HbA1c was measured by ion-exchange high-performance liquid chromatography. Total cholesterol, HDL cholesterol, and triglycerides were measured enzymatically, and creatinine by the Jaffé kinetic method at the National Institute of Nutrition laboratory.

### 2.6. Statistical Analysis

Continuous variables are summarized as mean ± sample standard deviation, and categorical variables as n/N (%), using available valid observations for each variable. Between-group comparisons used Welch’s t test for continuous variables, Pearson’s chi-square test for categorical variables, Fisher’s exact test for sparse 2 × 2 tables, and a conditional Monte Carlo chi-square test with 100,000 fixed-margin samples for larger sparse tables. Crude prevalence ratios (PRs) for DEB positivity were calculated for women versus men and for each HADS subscale ≥11 versus <11. PR confidence intervals used the log-Wald method:

$$\mathrm{SE}\left[\log(\mathrm{PR})\right]=\sqrt{\frac{1}{a}-\frac{1}{n_{1}}+\frac{1}{c}-\frac{1}{n_{0}}}$$

where a and c are DEB-positive counts, and n_1_ and n_0_ are the corresponding group totals. All tests were two-sided. Confidence intervals are 95%; *p*-values are exploratory, with no adjustment for multiple comparisons. Missing or invalid observations were excluded only from analyses requiring those variables; no imputation was performed.

The primary exploratory logistic model included age in years, female sex (versus male), BMI in kg/m^2^, diabetes duration in years, and PSS-4 score. These variables were selected for clinical relevance and to limit model complexity, without univariate *p*-value screening or stepwise selection. Loss of control, body-image items, fear of weight gain, eating symptoms, and other variables overlapping with the screening outcome were excluded from the adjusted models. Separate secondary models replaced PSS-4 with HADS-A or HADS-D and used the same four adjustment variables. Psychological scores were not entered together. Adjusted odds ratios (aORs) are reported per unit increase for continuous variables. The primary model used all 400 participants and 107 positive outcomes, with five slope parameters (21.4 events per parameter); the depression model used 399 participants.

For the primary model, variance inflation factors (VIFs) assessed collinearity. Linearity in the logit was examined by comparing each continuous linear term with a three-degree-of-freedom natural cubic spline, using likelihood-ratio tests. Model diagnostics included the area under the receiver operating characteristic curve (AUC), Brier score, and Hosmer–Lemeshow test, using deciles of estimated probability. Internal bootstrap assessment used 300 resamples to estimate AUC optimism and the average calibration slope when each bootstrap model was evaluated in the original sample.

## 3. Results

### 3.1. Demographic and Anthropometric Characteristics

Among 400 participants, 107 (26.8%; Wilson 95% CI, 22.6–31.3%) had DEB-positive status, and 293 were DEB-negative (Table 1). The mean age was 56.38 ± 8.21 versus 60.21 ± 8.86 years (*p* < 0.001), and mean BMI was 33.00 ± 7.77 versus 28.98 ± 5.80 kg/m^2^ (*p* < 0.001). Women accounted for 76.6% and 64.2% of the respective groups (*p* = 0.018). Using sex-specific denominators, DEB prevalence was 82/270 (30.4%) in women and 25/130 (19.2%) in men, giving a crude PR of 1.58 (95% CI, 1.06–2.35). Sedentary behavior, restrictive dietary advice, and reported alcohol use were more frequent in the DEB-positive group, although alcohol use was uncommon (eight participants overall). Dietary adherence was less frequent among DEB-positive participants. Table 1 provides further details.

### 3.2. Disordered Eating Behaviors, Patterns, and Psychological Profiles

DEB-positive participants more often reported loss of control over eating (82/107, 76.6%, versus 31/293, 10.6%) and snacking (68/107, 63.6%, versus 87/293, 29.7%; both *p* < 0.001; Table 2). Medication omission was recorded for 29/107 (27.1%) versus 46/293 (15.7%) participants (*p* = 0.010), and insulin dose reduction for 15/107 (14.0%) versus 19/293 (6.5%; *p* = 0.017). Bulimia, hyperphagia, and purging were more frequent in the DEB-positive group.

Perceived stress, anxiety, and depressive symptom scores were higher in the DEB-positive group (all *p* < 0.001). Table 2 reports further details.

With DEB positivity as the outcome, prevalence was 64/158 (40.5%) among participants with HADS-A ≥11 and 43/242 (17.8%) among those with HADS-A <11 (crude PR, 2.28; 95% CI, 1.64–3.17). For HADS-D ≥11 versus <11, the corresponding prevalence was 50/148 (33.8%) versus 57/251 (22.7%; crude PR, 1.49; 95% CI, 1.08–2.05).

### 3.3. Metabolic and Biochemical Parameters

Mean HbA1c was 10.36 ± 2.09% in 106 DEB-positive participants and 9.91 ± 2.01% in 290 DEB-negative participants (Table 3). The between-group difference was 0.45 percentage points (95% CI, −0.01 to 0.92; Welch *p* = 0.055). Both means indicate poor glycemic control. Total cholesterol was higher in the DEB-positive group (4.83 ± 1.09 versus 4.53 ± 1.18 mmol/L; *p* = 0.015). Differences in fasting glucose, HDL cholesterol, triglycerides, and creatinine were not statistically significant using the Welch tests.

### 3.4. Exploratory Adjusted Associations

**Figure 1:**
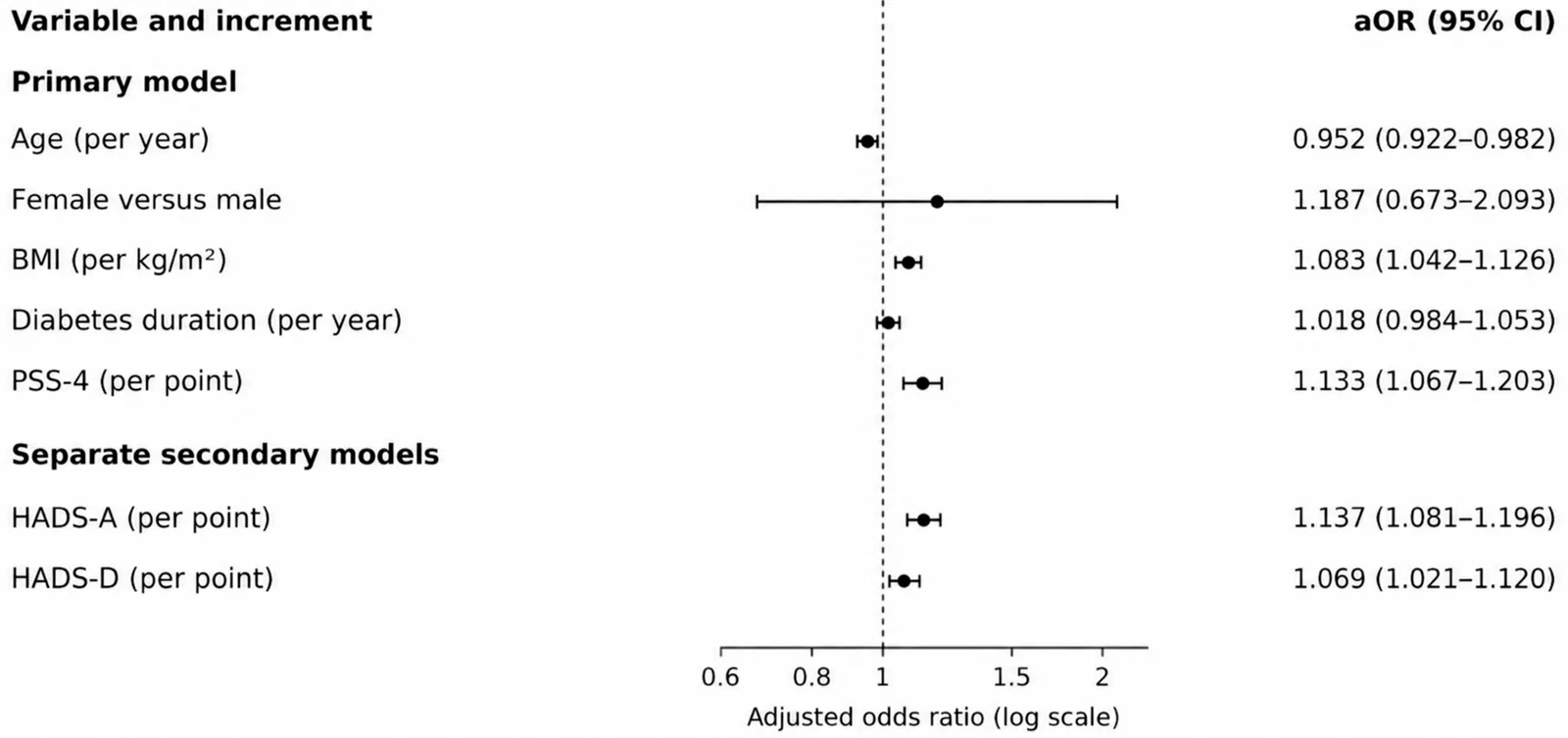
Adjusted associations with DEB screening positivity. Points show adjusted odds ratios (aORs); horizontal bars show 95% confidence intervals. The dashed line denotes an aOR of 1. The primary model includes age, sex, BMI, diabetes duration, and PSS-4 simultaneously (*N* = 400; 107 positive outcomes). Each secondary model replaces PSS-4 with the indicated HADS score and retains the four clinical covariates (HADS-A: *N* = 400; HADS-D: *N* = 399); psychological scores are not mutually adjusted. Male sex is the reference, and continuous-variable increments are indicated beside each row. Eating-symptom components of the outcome were excluded from adjustment.

In the primary model (Figure 1), each additional year of age was associated with lower odds of DEB positivity (aOR, 0.952; 95% CI, 0.922–0.982; *p* = 0.002). Higher BMI (aOR per kg/m^2^, 1.083; 95% CI, 1.042–1.126; *p* < 0.001) and PSS-4 score (aOR per point, 1.133; 95% CI, 1.067–1.203; *p* < 0.001) were associated with higher odds. Female sex and diabetes duration were not statistically significant after adjustment. In separate secondary models, the aOR per point was 1.137 (95% CI, 1.081–1.196; *p* < 0.001) for HADS-A and 1.069 (95% CI, 1.021–1.120; *p* = 0.004) for HADS-D.

Primary-model VIFs ranged from 1.03 to 1.23. Spline comparisons did not provide strong evidence against linearity (*p* = 0.591 for age, 0.190 for BMI, 0.720 for diabetes duration, and 0.306 for PSS-4). Apparent AUC was 0.736, bootstrap optimism-corrected AUC was 0.721, and Brier score was 0.168. The mean bootstrap test calibration slope was 0.922. The Hosmer–Lemeshow statistic was 5.93 with 8 degrees of freedom (*p* = 0.655).

## 4. Discussion

Approximately one-quarter of this Tunisian T2DM clinic sample had a positive DEB screen. We identified associations with younger age, higher BMI, and higher perceived stress after adjustment for selected clinical characteristics. Anxiety and depression scores were positively associated with DEB positivity in separate adjusted models.

### 4.1. Prevalence of Disordered Eating Behaviors in Type 2 Diabetes

Comparisons across studies require attention to outcome definition and population. Kenardy et al. reported regular binge eating in 20.9% of 215 women with T2DM using an Eating Disorder Examination assessment [17]. Papelbaum et al. reported an eating-disorder diagnosis in 20% of 70 patients with T2DM, with BED diagnosed in 10% [35]. In contrast, Allison et al. identified BED in 1.4% of 845 Look AHEAD applicants, using screening followed by interviews [46], and Mannucci et al. reported BED prevalence below 5% in each of three comparison groups of participants living with overweight or obesity [18]. A 2024 Indian study found that approximately 35% of 254 adults with T2DM screened as being at elevated eating-disorder risk [47]. Our 26.8% screening positivity cannot be directly equated with any of these diagnostic prevalence estimates. Diagnostic criteria, instrument choice, recruitment, and clinical characteristics all affect comparisons [48].

Women comprised a larger proportion of the DEB-positive group, consistent with sex differences reported in much of the general eating-disorder literature [49,50,51]. However, female sex was not statistically significant in the revised adjusted model, and patterns vary across T2DM samples [47]. Tunisian studies document substantial sex and socioeconomic differences in obesity [6,7], but obesity prevalence cannot establish eating-disorder prevalence. BED can occur across body sizes, and BMI alone is insufficient to identify clinically significant eating pathology [52,53].

Access to nutritious food is relevant to the interpretation of dietary recommendations. The World Food Program’s Tunisia country plan describes economic barriers to adequate diets [54], and national and regional nutrition-transition research documents changes in food consumption and obesity [8,55].

### 4.2. Age and Demographic Associations with Disordered Eating

General eating-disorder epidemiology describes age-related variation [56], while the Indian T2DM screening study also reported an inverse cross-sectional association with age [47]. These findings do not establish an age threshold for screening or imply that older adults are unaffected. Cohort effects, differences in reporting, and selection into specialist care are possible explanations that cannot be distinguished here.

### 4.3. Body Mass Index and Weight-Related Factors

Higher BMI was associated with DEB positivity in both descriptive and adjusted analyses. This is consistent with evidence that BMI and eating-related concerns can be related in T2DM [52,57], although cross-sectional data cannot establish the direction of the relationship. Loss of control, repeated dieting, and weight stigma are plausible considerations, but they should not be presented as mechanisms demonstrated by this study [25,53]. Individualized nutrition care can address metabolic goals alongside psychological well-being, with attention to the person’s eating patterns and experience of weight-related messages [58,59].

### 4.4. Perception of Dietary Restrictions and Diabetes Acceptance

Restrictive dietary advice was recorded more frequently among DEB-positive participants, while acceptance of diabetes and treatment adherence were less frequent. Dietary restraint has been discussed in models of binge eating [60], but diabetes nutrition guidance emphasizes individualized and sustainable plans rather than a uniform restrictive diet [59]. Diabetes distress is distinct from general stress and depression [32]; it was not measured with a diabetes-specific distress scale here. Poos et al. reviewed diabetes distress in people with type 1 diabetes who restricted insulin, a population different from this sample [61]. The study therefore cannot infer that restrictive advice or poor illness acceptance caused DEB, or that changing either factor would reduce its occurrence.

### 4.5. Loss of Control Eating

Loss of control was strongly associated with DEB status, but this finding is partly expected because SCOFF includes a loss-of-control item [36]. Such overlap can produce a strong association even in the absence of an independent etiological relationship. We therefore retained the item as a descriptive feature and excluded it, together with related eating-symptom and body-image variables, from adjusted models. BED and bulimia nervosa require a broader assessment of episodes, associated symptoms, duration, and compensatory behaviors [48,52]. A positive response to a loss-of-control question warrants further assessment rather than a diagnosis or a claim of a 27-fold future risk. Evidence about fasting and subsequent binge-eating symptoms from adolescent populations may inform hypotheses but does not establish the mechanism in adults with T2DM [62].

### 4.6. Psychological Comorbidity: Anxiety and Depression

Higher stress, anxiety, and depression scores accompanied DEB positivity, and each psychological score remained positively associated in a separate model adjusted for age, sex, BMI, and diabetes duration. The association is consistent with evidence of psychiatric comorbidity in eating-disorder populations [63,64,65] and with studies of psychological symptoms in diabetes clinics [33,34,66,67].

A recent systematic review of ten observational studies involving 2420 young people with type 1 diabetes found consistent associations between psychological distress and disordered eating [68]. The REDEEM trial showed that diabetes distress can improve with self-management and supportive interventions in adults with T2DM [69].

### 4.7. Metabolic Parameters and Glycemic Control

The estimated HbA1c difference was modest relative to the high levels in both groups. Its confidence interval included zero under Welch’s test, and the result was close to the conventional significance threshold. It should therefore be reported as an uncertain between-group difference rather than evidence that DEB disrupted glycemic control. Older T2DM studies have reported differing relationships between eating pathology and HbA1c [16,17,18]. In a more recent T2DM outpatient study, emotional and uncontrolled eating were associated with higher BMI, HbA1c, and triglycerides than a cognitive-restraint pattern after adjustment [70]. This supports clinical relevance while preserving the distinction between association and causation. Depression and glycemic control may also be related [34,71], but the present study cannot disentangle directionality, medication effects, or residual confounding. The cholesterol difference is similarly exploratory and was one of several biochemical comparisons.

### 4.8. Clinical Implications for Screening and Intervention

Current ADA guidance recommends validated screening for disordered or disrupted eating and review of treatment-related effects on hunger and intake, with specialist input when eating-disorder symptoms are present [72]. This is consistent with earlier psychosocial-care guidance and reviews of assessment in diabetes [9,73,74]. A brief questionnaire can initiate a clinical conversation, but positive screens require further evaluation and instruments need appropriate cultural and clinical validation. The French SCOFF study involved female students [75], and screening recommendations from type 1 diabetes research cannot be transferred uncritically to older T2DM populations [76]. The USPSTF evidence review found reasonable adult SCOFF accuracy but no studies directly evaluating the benefits and harms of screening [38]. Accordingly, this study supports attention to eating concerns within multidisciplinary care; it does not establish the effectiveness, optimal frequency, or outcome benefit of a particular screening program.

### 4.9. Study Limitations

The cross-sectional design prevents temporal or causal inference. Recruitment from one specialist clinic, the high HbA1c levels, and the eligibility restrictions limit generalizability. The Arabic BITE and SCOFF validations involved different populations, and local psychometric performance was not evaluated. The PSS-4 has known reliability limitations in some settings. Despite the exclusion of outcome-overlapping items and use of five slope parameters, unmeasured confounding and measurement error remain. Separate analyses of three psychological scales do not estimate mutually independent psychological effects, and multiple comparisons increase the possibility of chance findings. Model diagnostics and bootstrap estimates are internal assessments; they do not establish external validity. Single biochemical measurements, unmeasured treatment details, and the absence of longitudinal outcomes further limit interpretation.

## 5. Conclusions

In this specialist-clinic sample of 400 Tunisian adults with T2DM, 26.8% had a positive DEB screen. Younger age, higher BMI, and higher perceived stress were associated with DEB positivity in the exploratory model; anxiety and depressive symptoms were positively associated in separate adjusted analyses. HbA1c was high in both groups, and the modest between-group difference was uncertain. The findings support attention to eating concerns and psychological symptoms in diabetes care, with clinical assessment following positive screens. Prospective studies with diagnostic interviews and culturally appropriate measures are needed to clarify temporality and evaluate interventions.

## Figures and Tables

**Table 1:** Sociodemographic and anthropometric characteristics by DEB screening status.

Characteristic	DEB+ (*N* = 107)	DEB− (*N* = 293)	*p*-Value
Age (years)	56.38 ± 8.21	60.21 ± 8.86	<0.001
Female sex	82/107 (76.6)	188/293 (64.2)	0.018
Marital status: Married	82/107 (76.6)	226/293 (77.1)	0.083
Marital status: Unmarried	11/107 (10.3)	14/293 (4.8)	
Marital status: Widowed	14/107 (13.1)	53/293 (18.1)	
Education: No formal education	28/107 (26.2)	90/293 (30.7)	0.834
Education: Primary	49/107 (45.8)	128/293 (43.7)	
Education: Secondary	26/107 (24.3)	64/293 (21.8)	
Education: Higher	4/107 (3.7)	11/293 (3.8)	
Socioeconomic status: Lower	38/107 (35.5)	118/293 (40.3)	0.371
Socioeconomic status: Middle	66/107 (61.7)	160/293 (54.6)	
Socioeconomic status: Upper	3/107 (2.8)	15/293 (5.1)	
Employment: Unemployed	61/107 (57.0)	155/292 (53.1)	0.024
Employment: Employed	33/107 (30.8)	67/292 (22.9)	
Employment: Retired	13/107 (12.1)	70/292 (24.0)	
Alcohol use	5/106 (4.7)	3/293 (1.0)	0.034
Sedentary lifestyle	76/107 (71.0)	174/293 (59.4)	0.033
Dietary adherence	25/107 (23.4)	104/293 (35.5)	0.022
Restrictive dietary advice	41/107 (38.3)	58/293 (19.8)	<0.001
Daily snacks: 0	42/107 (39.3)	147/293 (50.2)	<0.001
Daily snacks: 1–2	56/107 (52.3)	142/293 (48.5)	
Daily snacks: ≥3	9/107 (8.4)	4/293 (1.4)	
BMI (kg/m^2^)	33.00 ± 7.77	28.98 ± 5.80	<0.001
BMI at least 25 kg/m^2^	93/107 (86.9)	220/293 (75.1)	0.011

DEB+, recorded positive screen; DEB−, recorded negative screen; BMI, body mass index. Continuous values are mean ± SD. Categorical values are n/N (%), with denominators reflecting available data. Categorical *p*-values compare the full distribution of each characteristic. Welch’s *t* tests, Pearson chi-square tests, Fisher’s exact test for alcohol use, and Monte Carlo chi-square tests for education, socioeconomic status, and snack categories were used. Percentages may not sum exactly to 100 because of rounding.

**Table 2:** Eating-related behaviors and psychological symptoms by DEB screening status.

Characteristic	DEB+ (*N* = 107)	DEB− (*N* = 293)	*p*-Value
Medication omission	29/107 (27.1)	46/293 (15.7)	0.010
Treatment adherence	77/107 (72.0)	252/293 (86.0)	0.001
Awareness that insulin affects weight	26/107 (24.3)	42/293 (14.3)	0.019
Insulin dose reduction	15/107 (14.0)	19/293 (6.5)	0.017
Diabetes acceptance	83/106 (78.3)	262/293 (89.4)	0.004
Bulimia	51/107 (47.7)	16/293 (5.5)	<0.001
Hyperphagia	4/107 (3.7)	0/293 (0.0)	0.005
Purging-related behavior	90/107 (84.1)	141/293 (48.1)	<0.001
Prandial hyperphagia	33/107 (30.8)	32/293 (10.9)	<0.001
Prandial hypophagia	16/107 (15.0)	76/293 (25.9)	0.021
Snacking	68/107 (63.6)	87/293 (29.7)	<0.001
Body image dissatisfaction	68/107 (63.6)	115/293 (39.2)	<0.001
Fear of weight gain	75/107 (70.1)	114/293 (38.9)	<0.001
Desire to lose weight	67/107 (62.6)	116/293 (39.6)	<0.001
Loss of control	82/107 (76.6)	31/293 (10.6)	<0.001
PSS-4 total	8.92 ± 4.03	6.60 ± 4.02	<0.001
HADS-A total	11.80 ± 4.96	8.32 ± 4.93	<0.001
HADS-D total	10.31 ± 5.48	8.13 ± 4.95	<0.001
HADS-A ≥11	64/107 (59.8)	94/293 (32.1)	<0.001
HADS-D ≥11	50/107 (46.7)	98/292 (33.6)	0.016

Values are n/N (%) or mean ± SD. **PSS-4:** Perceived Stress Scale, 4-item version, **HADS-A:** Hospital Anxiety and Depression Scale—Anxiety subscale, **HADS-D:** Hospital Anxiety and Depression Scale—Depression subscale.

**Table 3:** Biochemical measurements by DEB screening status.

Measurement	DEB+	DEB−	*p*-Value
HbA1c (%)	10.36 ± 2.09 (*N* = 106)	9.91 ± 2.01 (*N* = 290)	0.055
Fasting glucose (mmol/L)	11.88 ± 4.88 (*N* = 107)	11.03 ± 4.78 (*N* = 293)	0.127
Total cholesterol (mmol/L)	4.83 ± 1.09 (*N* = 107)	4.53 ± 1.18 (*N* = 292)	0.015
HDL cholesterol (mmol/L)	1.16 ± 0.29 (*N* = 107)	1.12 ± 0.28 (*N* = 292)	0.194
Triglycerides (mmol/L)	1.85 ± 0.98 (*N* = 107)	1.65 ± 1.15 (*N* = 291)	0.095
Creatinine (µmol/L)	61.97 ± 36.46 (*N* = 105)	64.81 ± 34.71 (*N* = 292)	0.490

Values are mean ± SD, with group-specific available *N* shown. All *p*-values are from two-sided Welch’s *t* tests. HbA1c, glycated hemoglobin; HDL, high-density lipoprotein.

## Data Availability

The dataset is publicly available in Figshare under the title “Eating disorder and diabetes”, deposited by Rym Ben Othman in 2023. It is available at https://doi.org/10.6084/m9.figshare.24887991 accessed on 21 December 2023 v1 under a CC BY 4.0 license.
